# An evaluation of patient-reported outcomes in sickle cell disease within a conceptual model

**DOI:** 10.1007/s11136-022-03132-z

**Published:** 2022-04-21

**Authors:** Marsha J. Treadwell, Swapandeep Mushiana, Sherif M. Badawy, Liliana Preiss, Allison A. King, Barbara Kroner, Yumei Chen, Jeffrey Glassberg, Victor Gordeuk, Nirmish Shah, Angela Snyder, Theodore Wun, For the Sickle Cell Disease Implementation Consortium (SCDIC)

**Affiliations:** 1 University of California San Francisco Benioff Children’s Hospital Oakland, 747 52nd Street, Oakland, CA 94609 USA; 2 Medical College of Georgia - Augusta University, Augusta, GA USA; 3 Anne & Robert H. Lurie Children’s Hospital of Chicago, Chicago, IL USA; 4 Northwestern University Feinberg School of Medicine, Chicago, IL USA; 5 Research Triangle International, Research Triangle Park, NC USA; 6 Washington University St. Louis, St. Louis, MO USA; 7 Mount Sinai Hospital, New York, NY USA; 8 University of Illinois Chicago, Chicago, IL USA; 9 Duke University, Chapel Hill, NC USA; 10 Georgia State University, Atlanta, GA USA; 11 University of California Davis, Sacramento, CA USA

**Keywords:** Patient-reported outcome measures, Sickle cell disease, Implementation science, Models—biopsychosocial

## Abstract

**Purpose:**

To examine the relations between patient-reported outcomes (PROs) within a conceptual model for adults with sickle cell disease (SCD) ages 18 – 45 years enrolled in the multi-site Sickle Cell Disease Implementation Consortium (SCDIC) registry. We hypothesized that patient and SCD-related factors, particularly pain, and barriers to care would independently contribute to functioning as measured using PRO domains.

**Methods:**

Participants (*N* = 2054) completed a 48-item survey including socio-demographics and PRO measures, e.g., social functioning, pain impact, emotional distress, and cognitive functioning. Participants reported on lifetime SCD complications, pain episode frequency and severity, and barriers to healthcare.

**Results:**

Higher pain frequency was associated with higher odds of worse outcomes in all PRO domains, controlling for age, gender and site (OR range 1.02–1.10, 95% CI range [1.004–1.12]). Reported history of treatment for depression was associated with 5 of 7 PRO measures (OR range 1.58–3.28 95% CI range [1.18–4.32]). Fewer individual barriers to care and fewer SCD complications were associated with better outcomes in the emotion domain (OR range 0.46–0.64, 95% CI range [0.34–0.86]).

**Conclusions:**

Study results highlight the importance of the biopsychosocial model to enhance understanding of the needs of this complex population, and to design multi-dimensional approaches for providing more effective interventions to improve outcomes.

**Supplementary Information:**

The online version contains supplementary material available at 10.1007/s11136-022-03132-z.

## Plain English summary

Sickle cell disease (SCD) is a rare, inherited blood disorder that causes serious and life-threatening complications including pain, stroke and anemia. It is important to understand the burden of the disease, particularly as patients get older, but there are few studies in this area. Patient-reported outcomes (PROs) communicate information about aspects of patients’ lives that can only be provided from their point of view. Our goal was to examine how PROs were inter-related with pain and other sickle cell complications, barriers to care and other social variables for adults with SCD. We gained valuable insights into the impact of pain, depression, employment and income on quality of life for adults with SCD, as measured by the PROs in our study, and we showed how important barriers to care can be. We contributed to the knowledge base about PRO measurement in SCD. PRO measures thus provide meaningful information for providers and patients to improve quality of life, and the most effective interventions to improve health outcomes must be multi-dimensional.

## Introduction

The routine assessment of patient-reported outcomes (PROs) in clinical settings lends to creating a patient-centered environment, by enhancing communication and shared-decision making, improving satisfaction, and allowing for monitoring improvement or deterioration of health status [1]. In clinical trials, PROs complement measures of efficacy such as survival and healthcare utilization, allowing translation of results into some benefits that can only be evaluated with patients’ reports.

Sickle cell disease (SCD) is a rare, inherited blood disorder in the U.S., affecting about 100,000 individuals, primarily African Americans [2]. The clinical manifestations of the disease include recurrent, unpredictable and severe acute pain episodes; chronic pain; cerebrovascular disease, including overt stroke; and other serious complications such as renal and cardiopulmonary disease. These manifestations can lead to significant impairment as assessed using PROs [3, 4], and an increased burden of disease as patients age [5].

The National Heart, Lung and Blood Institute (NHLBI), in collaboration with a range of sickle cell stakeholders, developed the Adult Sickle Cell Quality of Life Measurement Information System (ASCQ-Me®) to provide a means of systematically evaluating disease-specific PRO domains impacted for the growing population of adults with SCD [6]. The ASCQ-Me development used advanced knowledge of psychometrics as it aligned with the development of the National Institutes of Health’s Patient-Reported Outcomes Measurement Information System (PROMIS®).

Previous research has shown that adults and children with SCD who received the disease modifying therapy hydroxyurea, compared to those who did not, reported better PROs [7–10]. Demographic factors associated with worse PRO scores for adults with SCD include age and sex [11, 12]. Other factors associated with worse PRO scores include SCD complications, particularly pain [13, 14].

As lifespans have increased, disparities in quality of life and quality of care are also evident for adults with SCD, including lower socioeconomic status [15, 16], stigma, discriminatory treatment in healthcare settings [17], lack of social support, isolation, and cognitive challenges [6, 18–20]. The prevalence of depression and anxiety is two to three times the national average [21].

A few studies have delineated how this range of challenges, manifested by scores on PROs measures, are associated with healthcare utilization. For example, lower education was found to be independently associated with potentially avoidable emergency department (ED) care [22]. Young adults with worse PRO scores evidenced more frequent SCD-related hospitalizations and ED visits and/or longer hospitalizations [7, 22, 23]. The three-year Comprehensive Sickle Cell Centers (CSCC) Collaborative Data Project that began in 2005 included 1046 participants (median age 28.0 years, 48% male, 73% SS or Sβ^0^ thalassemia) [5]. Participants reported impaired health related quality of life (HRQoL) on all but the mental health domain on the SF-36, particularly with increasing age [5]. Pain episodes, asthma, or avascular necrosis were associated with worse SF-36 scale scores as was chronic opioid use. Female gender was associated with impaired physical function and vitality scale scores and chronic antidepressant use was associated with worse scores on bodily pain, vitality, social functioning, emotional role, and mental health scales. Few studies have examined the inter-relations between PROs and different patient- and/or SCD-related variables within a conceptual model. Conceptual models may allow us to advance our understanding of how the disease and treatments affect individuals, as more treatment options become available.

The Sickle Cell Disease Implementation Consortium (SCDIC) was established by NHLBI in 2016 to identify and address barriers to quality care in SCD [24]. A key activity was to engage eight sites in diverse regions in the U.S. to create a registry of a minimum of 2400 adolescents and adults with SCD. The goal of the SCDIC registry is to enhance our understanding of SCD acute and chronic complications/comorbidities and treatments, as well as HRQoL and PROs for a modern cohort. We recently described the SCDIC registry methodology [25] and the preliminary evaluation of PROs in this population [26].

**Fig. 1:**
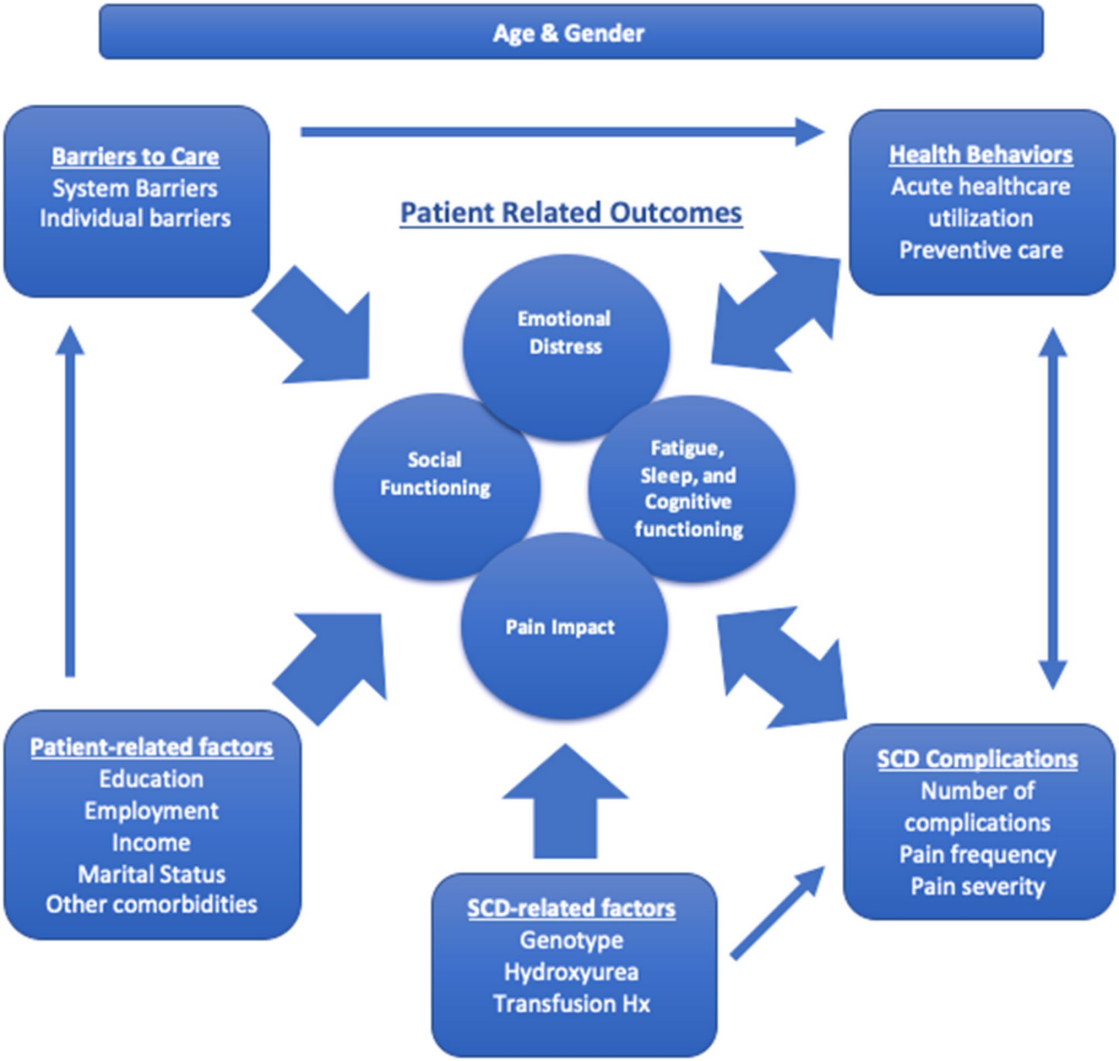
Conceptual model for inter-relations of patient-reported outcomes (PROs) in sickle cell disease (SCD). The model includes the inter-relations of four PRO groups (emotional distress, social functioning, pain impact, and fatigue, sleep and cognitive functioning) with health behaviors (acute healthcare utilization and preventive care), SCD complications (number of complications and pain frequency/severity), SCD-related factors (genotype, hydroxyurea, chronic transfusion history), patient related factors (education, employment, income, marital status, diabetes and depression) and barriers to care (systemic and individual). All inter-relations are adjusted for age and gender identity

The purpose of the present analysis is to examine the relations between PROs within a conceptual model for adults with SCD ages 18–45 years enrolled in the SCDIC registry. The PRO domains assessed—emotional, pain, fatigue and sleep impacts, and social and cognitive functioning—are influenced by SCD complications, disease modifying therapies, socio-demographics, barriers to care and healthcare utilization. The conceptual model was developed from prior formative research [6], and from the SCDIC's Conceptual Framework for PROs/HRQoL in SCD [27], with input from other national and international experts in the area of PROs in SCD. Figure 1 shows the inter-relations between different variables along with their impact on PROs. Our conceptual model reflects prior research on the inter-relations among variables such as barriers to care [28], utilization patterns [29], morbidity [30], and socio-demographic factors [31, 32] and health outcomes. We hypothesized that patient and SCD-related factors as well as barriers to care would independently contribute to functioning as measured using the PRO domains. We expected that the experience of pain and other SCD-related complications would account for a significant degree of the relation between the variables.

## Method

### Participants and procedures

Adolescents and adults with SCD ages 15–45 years were enrolled in the SCDIC registry during an 18-month period between 2017 and 2018. Inclusion criteria was confirmed SCD diagnosis (SS, SC, Sβ-thalassemia, other variants), literate in English, and willing and cognitively able to provide informed consent and complete the Patient Enrollment Survey. SCD diagnosis was confirmed through medical record or confirmatory laboratory test. Individuals with sickle cell trait (e.g., Hb AS), successful bone marrow transplant for SCD, or unwilling/unable to provide consent were excluded. Our sub-sample included 2,054 adults 18—45 years and with completed Patient Enrollment Surveys and Medical Record Abstraction forms.

The study utilized convenience sampling from the eight SCDIC sites, with some outreach into the community. Eligible participants were identified and recruited in-person (e.g., clinic, outreach events), by phone, or via electronic media (e.g., websites, chat rooms). Participants recruited remotely provided verbal informed consent and submitted signatures online. SCDIC research staff were available to answer questions as needed while participants completed the surveys. A member of the local study team completed a medical record abstraction for each participant. Institutional review boards of all SCDIC sites provided approval, with seven of the eight sites providing compensation for participation. All data were collected at one point in time. Details of study methodology were recently published [26].

### Measures

Participants completed a 48-item survey including socio-demographics and PRO measures. We did not use complete short forms for some ASCQ-Me® and PROMIS® measures to reduce participant burden. However, the PROs that were selected were developed using item response theory (IRT), that allows for a range of administration and tailoring options [33]. An IRT-calibrated item bank consists of items that correspond with level of symptom severity or function. Any number and combination of items from the same bank can be scored and compared to all other measures derived from the same item bank without loss of precision in measurement of the construct [34]. Individual items were selected for their relevance from domains by the SCDIC investigators. Of note, interpretation of scale scores must take into consideration that the reference populations for ASCQ-Me® and PROMIS® differ, with the former consisting of adults with SCD and the latter adults from the general population. Thus, the “average” score of 50 for the PROMIS measures centers on a sample of individuals that, collectively, matched the U.S. 2000 Census on such demographics as gender, age, race/ethnicity and education, while the “average” score of 50 for the ASCQ-Me measures centers on a sample of adults with SCD 18 years of age and older.

ASCQ-Me® Emotional Impact over the past seven days was assessed using: How often did you … “feel completely hopeless because of your health?” and “were you very worried about needing to go to the hospital?” ASCQ-Me® Social Functioning over the past 30 days was assessed with: How much … “did you rely on others to take care of you because of your health?” and “did your health make it hard for you do things with your friends?” ASCQ-Me® Pain Impact over the past seven days was assessed using: How often … “did you have very severe pain?” and “did you have pain so bad that it was hard to finish what you were doing?” ASCQ-Me® Sleep Impact over the past seven days was assessed using: How often did you … “stay up most of the night because you could not fall asleep?” and “have a lot of trouble falling asleep?” All ASCQ-Me items were scored on a 5-point Likert scale (i.e., Never to Always). Item responses were uploaded to HealthMeasures Scoring Service at assessmentcenter.net, where T-scores and related statistics were generated, using adults with SCD who participated in the ASCQ-Me field test (*n* = 555) as the reference population [14]. The standardized T-score mean is 50 (standard deviation (SD) = 10), with higher scores indicating better outcomes.

The ASCQ-Me® Pain Episode question set includes five questions regarding the frequency (number of severe pain events in the last 12 months), timing (of most recent event) and severity of the most recent pain event (duration and pain interference). A Pain Episode composite was calculated by creating standard scores for the pain episode frequency and severity composites. A higher score indicates worse frequency, timing and severity of SCD pain.

Cognitive functioning over the past seven days was assessed using the 8-item Neuro-QOL Cognitive Function short form [35] with item responses on a 5-point Likert scale (i.e., Never to Very Often). Item responses were uploaded to the HealthMeasures Scoring Service, where T-scores and related statistics were generated using PROsetta Stone Wave 2 as the reference population, which is representative of the general adult population [36]. A higher T-score indicates better cognitive function.

The 4-item, PROMIS® short form for Emotional Distress-Depression was used to assess depressive symptoms over the past seven days and items were scored on a 5-point Likert scale (i.e., Never to Always). Crosswalk tables have been established using rigorous methodology, to link such “legacy” depression measures as the Patient Health Questionnaire (PHQ)-9, the Beck Depression Inventory-II (BDI-II) and the Center for Epidemiologic Studies Depression Scale (CES-D) with the PROMIS® Emotional Distress-Depression measure [37]. Analyses have shown that PROMIS cutoff scores for depression severity correspond with commonly used legacy measures [38].

A single item (“I felt tired”) from the PROMIS Fatigue item bank was used to measure tiredness in the past seven days, on a 5-point Likert scale from Not at All to Very Much. Item responses were uploaded to the HealthMeasures Scoring Service, where T-scores and related statistics were generated using PROMIS Wave 1 as the reference population, which is representative of the general adult population [39]. Higher T-scores on these PROMIS measures indicate worse outcomes.

Participants reported on lifetime SCD complications using the ASCQ-Me Medical History Checklist (MHC) [14], modified and expanded by the SCDIC investigators from the original list of nine to include 13 treatments and conditions associated with SCD, answered “yes” or “no.” Treatments included daily pain medicine and conditions included lung problems (e.g., acute chest syndrome); kidney, eye, hip or shoulder damage; asthma; pulmonary hypertension; heart failure; blood clots; stroke; leg ulcers; and spleen damage or removal. The score for the checklist is the number of questions with a “yes” response, thus a higher score indicates a greater number of treatments or conditions. Participants reported separately on two comorbidities—diabetes (“yes” or “no”) and current or ever treated for depression. They indicated their current use of disease modifying therapies hydroxyurea and/or regular blood transfusions (“yes” or “no”).

The SCD Barriers to Medical Care consists of 11 reasons for experiencing delays or not receiving needed medical care (“yes” or “no,” grouped into seven Access Barriers (e.g., distance from provider, insurance, challenges obtaining an appointment) and four Individual Barriers (e.g., too busy, previous bad experiences with the healthcare system) [12].

Finally, we tracked healthcare utilization (an aspect of "Health Behaviors" shown in Fig. 1) utilizing (1) outpatient visit with sickle cell specialist or primary care provider within one year of enrollment (“yes” or “no”) and (2) number of acute care visits for pain in the past year, categorized as 0, 1–2 or >  = 3 ED visits or hospital admissions, both from medical record abstraction.

### Statistical analyses

All analyses were performed on cross-sectional data, with seven PROs (i.e., pain, sleep, emotional and social functioning impacts, emotional distress, tiredness, and cognitive function) included in the analyses. Binary variables were created for all outputs using as cut-points one standard deviation (SD) above the mean for PROs where a higher T-score indicates a worse outcome (PROMIS® Emotional Distress and Fatigue) [41] and one SD below the mean for those where a higher T-score indicates a better outcome as compared to the reference populations (ASCQ-Me® and Neuro-QoL Cognitive Function) [32].

Baseline characteristics and distributions of risk factors are presented as frequencies and percentages for categorical variables, and median and interquartile ranges (IQR) or mean and SD for continuous variables. Categorical variables were analyzed using chi-square, or Fisher’s exact test for sparse tables. Continuous variables were compared using t-test or Mann–Whitney U test, as appropriate.

Univariate analysis was used to evaluate potentially significant variables for inclusion in multivariable models for each PRO to identify factors independently associated with better or worse outcomes. Variables with *p* <  = 0.10 in univariate analysis were included in a multivariable logistic regression with backward elimination. Adherence with hydroxyurea and healthcare utilization (ED and inpatient visits) were not included in the models because not all participants were eligible for or prescribed hydroxyurea and about 27% of records were missing data on utilization. Age, gender and site were included and retained in all models during stepwise reduction regardless of their statistical significance to control for potential confounding effects. To account for multiple testing, a *p*-value = 0.01 was used as the threshold for statistical significance in the multivariable models. Odds ratios (OR) and corresponding 95% confidence intervals were obtained for variables remaining in the final model. All analyses were conducted in SAS Version 9.4 (SAS Institute Inc., Cary, NC, USA).

## Results

### Socio-demographics

The median age of the 2054 adults with SCD in this analysis was 28 years (Table 1), and the predominant age group was 24–34 years (43.8%). Over half (56.8%) identified as female, and the majority identified as African American/Black (95.7%), with 4.5% reporting Hispanic ethnicity. The most common educational attainment was some college (35.2%), followed by high school graduate or equivalent (30.3%), with 24.1% attaining a college or advanced degree. Over a third (37.2%) were employed, 25.2% reported being disabled and the remainder were not working, either due to student status (13.5.%), or “other” (24.1%, e.g., maintaining their home or laid off). A significant proportion (74.2%) were never married and over half (54.6%) reported an annual income under $25,000, while the mean household density was 3. Like other SCD populations, almost 60% had Medicaid or other government-sponsored insurance.

**Table 1:** Participant socio-demographics

Characteristic	N = 2054
*Age*	
Mean (SD) years	29.1 (7.2)
Median (IQR)	28 (23–35)
	*n* (%)
18 to 24 years	641 (31.2)
25 to 34 years	900 (43.8)
35 to 45 years	513 (25.0)
*Gender*	
Male	888 (43.2)
Female	1166 (56.8)
*Race/Ethnicity*	
Black/African American	1918 (95.7)
Multi-racial	67 (3.3)
Other Race (American Indian/Alaska Native, Asian, White)	20 (0.9)
Hispanic ethnicity	91 (4.5)
*Highest Education*	
Some high school or less	209 (10.4)
High School (Graduate, GED or equivalent)	612 (30.3)
Some college	711 (35.2)
College graduate or advanced degree	487 (24.1)
*Employment*	
Working now	748 (37.2)
Disabled	507 (25.2)
Student	272 (13.5)
Other (unemployed, retired)	485 (24.1)
*Marital status*	
Married or living together	313 (16.2)
Never married	1499 (77.6)
Not married (divorced/separated, widowed)	120 (6.2)
*Insurance*	
Medicaid, other government-sponsored	1216 (59.2)^a^
Private	567 (27.6)
Medicare	468 (22.8)
None	83 (4.0)
Other	16 (0.8)
*Annual household income*	
$25,000 or less	998 (54.6)
$25,000—$50,000	403 (22.1)
$50,001 or more	426 (23.3)
Household density Mean (SD)	

^a^Percentages add up to greater than 100% as more than one option could be selected

### Clinical characteristics, health behaviors and barriers to care

The majority (72.6%) of participants were diagnosed with sickle cell anemia (SCD genotypes SS or Beta 0 thalassemia—Table 2). Of thirteen potential SCD treatments/complications, participants reported a median of 3 treatments/complications on the ASCQ-Me MHC with 38.7% reporting three or more treatments/complications, 38.7% reporting 2—3 and 22.6% reporting 0—1 SCD-related treatments/complications. Less than three percent reported a diagnosis of diabetes, and 26% reported current or previous treatment for depression. Forty-eight percent were currently using hydroxyurea and 28.8% were currently receiving regular blood transfusions. Over 80% of participants reported no barriers to needed healthcare, with 18.2% reporting 1 or more Access barriers and 18.1% reporting 1 or more Individual barriers*.* Almost all (92.2%) participants had outpatient visits with their primary care provider or SCD specialist within the past year. More than half (55.2%) had three or more ED or inpatient admissions for acute pain episodes in the past year (27% missing data). For ASCQ-Me® Pain Episode Frequency and Severity T-scores, means and standard deviations were similar to the reference sample, with a pain episode frequency mean (SD) of 49.2 (11) and pain severity mean (SD) of 50.8 (9.7).

**Table 2:** Clinical characteristics, barriers to care and health behaviors

Characteristic	N = 2054
From patient survey	
*ASCQ-Me Medical History Checklist*	
Median (IQR)	3 (2)
Range	0–12
Sickle cell disease diagnosis	n (%)
Hb SS or Sβ^0^ thalassemia	1490 (72.6)
Hb SC	432 (21.1)
Hb Sβ + thalassemia and other variants	130 (6.3)
Diabetes	
Yes	53 (2.6)
No	1953 (97.4)
Ever treated for depression	
Yes, current	181 (9.2)
Yes, previous	330 (16.8)
No	1455 (74.0)
Hydroxyurea use and adherence	
Yes, adherent (6–7 of 7 days)	628 (31.3)
Yes, partially adherent (2–5 of 7 days)	250 (12.5)
Yes, not adherent (0–1 of 7 days)	91 (4.5)
No, not currently using	1035 (51.7)
Regular blood transfusions for SCD	
Yes	587 (28.8)
No	1449 (71.2)
Barriers to Care	
*Access/Accommodations/Insurance*	
No barriers	1681 (81.8)
1–2 barriers	302 (14.7)
3 or more barriers	71 (3.3)
Individual barriers	
No barriers	1683 (81.9)
1–2 barriers	323 (15.7)
3 or more barriers	48 (2.3)
From Medical Record Abstractions:	
*Outpatient visit to hematologist or primary care provider within past 12 months*	
Yes	1893 (92.2)
No	92 (4.5)
Unknown	69 (3.4)
Emergency department (ED) and inpatient admissions for pain within past 12 months	
No ED or inpatient admissions	279 (18.7)
1–2 ED or inpatient admissions	388 (26.0)
3 or more ED or inpatient admissions	823 (55.2)

^a^Patients with missing data are not included in calculations of percentages unless otherwise specified

### Patient-reported outcomes: multivariable models

On the ASCQ-Me® measures, means and standard deviations were similar to the reference sample. Using the Emotional and Social Functioning Impact measures, participants reported mean (SD) scores of 50.5 (8.8) and 51.2 (9.7) respectively and only a few reported T-scores less than 40, with 12.7% reporting worse emotional impact and 14.8% reporting worse social functioning compared to the population norms. Somewhat higher percentages of participants reported T-scores less than 40 for Pain (mean (SD) of 47.1 (9.0)) and Sleep Impact (mean (SD) of 49.2 (9.7)), with 21.5% reporting worse impact of pain and 16.9% reporting worse sleep impact. For Neuro-Qol Cognitive Functioning, the mean (SD) was 50.3 (9.1) with 12.9% of the sample reporting impaired cognitive functioning (T-score < 40). For PROMIS Emotional distress, the mean (SD) was 50.9 (9.6), with 20.1% reporting worse emotional distress (T-score > 60). Finally, for PROMIS® Fatigue (tiredness), the mean (SD) was 55.4 (9.5) with 22% reporting worse tiredness (T-score > 60).

Results for univariate models can be found in supplemental materials (Table S1). Based on these results, age, gender, income, employment status, marital status, ever treated for depression, access and individual barriers to care, pain frequency and severity, and number of reported complications were entered in the multivariable models according to our selection criteria for each outcome.

In the multivariable model for Emotional Impact (Table 3) ever treated for depression, and pain frequency and severity were associated with higher odds for worse outcomes, while fewer individual barriers to care and fewer than three complications on the MHC were associated with better outcomes on Emotional Impact. Employment (disabled or “other” status), ever treated for depression, and pain frequency and severity were associated with higher odds for worse social functioning in the multivariable model for Social Functioning Impact, while fewer individual barriers to care were associated with lower odds of worse social functioning impact. In the model for Pain Impact, disabled and “other” employment status and higher pain frequency/severity was associated with higher odds of poor outcomes. In the model for Sleep Impact, ever treated for depression, income less than $50,000, and increased pain frequency and severity were associated with worse outcomes while fewer than three complications on the MHC were associated with lower odds for poor outcomes. For Neuro-QoL® Cognitive Function, ever treated for depression and income of $25,000 and less were associated with higher odds for worse cognitive functioning, while fewer access barriers to care were associated with lower odds for poor cognitive functioning.

**Table 3:** Significant multivariable relations between patient-reported outcomes and demographic and clinical characteristics

Model	Predictor	OR (95% CI)
ASCQ-Me® Emotional Impact		
	Ever treated for depression	2.30 (1.67–3.17)**
	# Individual barriers to care	
	0 versus 1 or more	0.50 (0.35–0.70)**
	ASCQ-Me® Pain Frequency	1.05 (1.03–1.07)**
	ASCQ-Me® Pain Severity	1.07 (1.04–1.09)**
	ASCQ-Me® SCD-MHC	
	Low (0–1)	0.54 (0.33–0.86)**
	Medium (2–3)	0.49 (0.34–0.70)**
	High (> 3)	Ref.
ASCQ-Me® Social Functioning Impact		
	Employment	
	Disabled	3.65 (2.50–5.39)**
	Student	1.49 (0.87–2.51)
	Other	2.40 (1.62–3.58)**
	Working	Ref.
	Ever treated for depression	1.58 (1.18–2.13)*
	# Individual barriers to care	
	0 versus 1 or more	0.54 (0.39–0.75)**
	ASCQ-Me® Pain Frequency	1.04 (1.03–1.06)**
	ASCQ-Me® Pain Severity	1.08 (1.06–1.11)**
ASCQ-Me® Pain Impact		
	Employment	
	Disabled	2.31 (1.68–3.2)**
	Student	1.05 (0.66–1.66)
	Other	2.05 (1.48–2.84)**
	Working	Ref.
	ASCQ-Me® Pain Frequency	1.10 (1.08–1.12)**
	ASCQ-Me® Pain Severity	1.10 (1.08–1.12)**
ASCQ-Me® Sleep Impact		
	Income	
	$25,000 and under	1.92 (1.3–2.89)**
	$25,001—$50,000	1.95 (1.24–3.1)**
	$50,001 +	Ref.
	Ever treated for depression	2.10 (1.56–2.81)**
	ASCQ-Me® Pain Frequency	1.03 (1.01–1.04)**
	ASCQ-Me® Pain Severity	1.03 (1.01–1.05)**
	ASCQ-Me® SCD-MHC	
	Low (0–1)	0.43 (0.27–0.67)**
	Medium (2–3)	0.65 (0.48–0.89)*
	High (> 3)	Ref.
Neuro-QoL™ Cognitive Functioning		
	Income	
	$25,000 and under	2.03 (1.36- 3.21)**
	$25,001—$50,000	1.57 (0.97- 2.57)
	$50,001 +	Ref.
	Ever treated for depression	2.18 (1.61–2.93)**
	# Accessl barriers to care	
	0- versus 1 or more	0.57 (0.41–0.79)**
PROMIS® Emotional Distress		
	Income	
	$25,000 and under	1.97 (1.39–2.85)**
	$25,001—$50,000	1.36 (0.88–2.09)
	$50,001 +	Ref.
	Ever treated for depression	3.28 (2.50–4.32)**
	# Individual barriers to care	
	0- versus 1 or more	0.46 (0.34–0.63)**
	ASCQ-Me® Pain Frequency	1.02 (1.01–1.04)**
	ASCQ-Me® SCD-MHC	
	Low (0–1)	0.62 (0.41–0.91)**
	Medium (2–3)	0.64 (0.47–0.86)**
	High (> 3)	Ref.
PROMIS® Fatigue/Tiredness		
	Gender Identity Male	0.38 (0.29–0.49)**
	Ever treated for depression	1.87 (1.47–2.39)**
	# Access barriers to care	
	0 versus 1 or more	0.47 (0.36–0.62)**
	ASCQ-Me® Pain Severity	1.02 (1.004–1.03)*

ASCQ-Me®: Adult Sickle Cell Quality of Life Measurement Information System

ASCQ-Me® SCD-MHC: ASCQ-Me®: Sickle Cell Disease Medical History Checklist

Neuro-QoL™: Quality of Life in Neurological Disorders

PROMIS®: Patient-Reported Outcomes Measurement Information System

*p < .01

**p < .001

^a^All models were adjusted for gender, age group, and site. ORs for these variables were included in the table only when statistically significant

In the multivariable model for PROMIS Emotional Distress, incomes of $25,000 and less, ever treated for depression and higher pain frequency were associated with higher odds for worse outcomes, while fewer individual barriers to care and fewer than three complications on the MHC were associated with lower odds for poor outcomes. Finally, in the model for PROMIS Fatigue (tiredness), ever treated for depression and higher pain severity were associated with higher odds for worse reports of tiredness, while male gender and fewer access barriers to care were associated with lower odds for tiredness.

## Discussion

We hypothesized that patient and SCD-related factors as well as barriers to care would independently contribute to functioning as measured using PRO domains from the ASCQ-Me®, PROMIS® and Neuro-QoL™ measurement systems. We expected that the experience of pain and other SCD-related complications would account for a significant degree of the relation between the variables and the PRO domains. Generally, our findings were consistent with study hypotheses, with higher pain frequency and history of treatment for depression associated with higher odds of worse outcomes in almost all PRO domains studied, with findings remaining when controlling for age, gender and site. Such socio-demographic variables as lower household income and unemployment, particularly due to disability status, were also associated with higher odds of worse outcomes on some of the PRO domains. Our study includes consideration of barriers to care, and we found that reports of fewer individual barriers to care were associated with better outcomes on measures in the emotion domain. We also found that fewer self-reported SCD complications/treatments were associated with better outcomes in the emotion domain.

Our findings are consistent with previous research [5, 42, 43] that highlighted dimensions of pain experiences associated with worse outcomes on PROs for adults with SCD, as well as depression [44, 45]. However, our study includes the first large, multi-site cohort of adults with SCD who completed contemporary PRO measures that have been developed and validated with state-of-the-science psychometric methods. We thus contribute to the accumulation of information on the precision, applicability and interpretation of these next generation measurement systems.

Reports on the PRO measures for our study participants were on average similar to reference samples, although with considerable variability within and across domains. For several domains, about 20% of participants of large-scale PROMIS reference samples have demonstrated moderate to severe symptomatology or functional impairment [46, 47]. About 20% of participants in the current study reported moderate/severe pain, emotional impact or tiredness. However, the proportion of participants with moderate/severe emotional distress (12.7%) and social functioning (14.8%) and sleep impact (16.9%) on ASCQ-Me were less than that seen in the reference population for PROMIS. In the current study, the two measures therefore appear to be assessing different constructs, in contrast with a recent study including 42 adults who demonstrated severe impairments on most domains assessed on both ASCQ-Me and PROMIS Global Health measures [42]. The Esham sample also experienced more severe and more frequent pain episodes compared with the ASCQ-Me reference sample and the timing of administration of the PROs occurred in relation to hospital admissions, while the SCDIC Registry participants completed the measures as outpatients.

Approximately 13% of respondents fell below the moderate/severe threshold on the Neuro-QOL Cognitive Function short form [35] which measures concerns about general cognition and executive function [48, 49]. The multivariable model was consistent with prior studies in that worse reports of cognitive function were associated with depressive symptoms and with lower incomes [50, 51]. Given that multiple cognitive domains have been shown to be increasingly negatively affected across the lifespan for the SCD population, this is an area of particular importance for future research [52]. Further exploration is also needed of validity, reliability, interpretability, and responsiveness of scores from the SCD specific ASCQ-Me measures and the comprehensive measurement systems including PROMIS and Neuro-QOL using large, multi-site samples [40].

Complex relations were also found among measures. Frequency of pain and history of depression were associated with the highest odds for worse emotional and sleep impacts, consistent with other studies [53, 54]. Pain experiences combined with unemployment (particularly related to disabled status) played a significant role in worse outcomes on social functioning. We consistently found that fewer patient reports of SCD-related complications and treatments were associated with better outcomes on the PRO measures. Thus, when considering clinical and research interventions, there is ample evidence that HRQoL in SCD must be viewed as a complex biopsychosocial phenomenon and there is a need for specific focus on pain experience and depression.

While disparities in HRQoL and quality of care are well-recognized in SCD, particularly for adults [55, 56], the impact of barriers to care has not been widely studied. We used a modified version of the first disease-specific measure of barriers to care in SCD and demonstrated that most participants in the SCDIC registry reported no barriers to needed care, and fewer barriers to care were associated with better outcomes on all PRO measures except pain and sleep impact. In a recent study of 303 adults with SCD, and in the SCDIC needs assessment with over 400 adolescents and adults with SCD, the most reported barriers to receiving care were costs, and perceived discrimination by and mistrust in healthcare professionals [32, 57].

## Limitations

Despite participation from multiple sites across the U.S., the generalizability of the sample may still represent a limitation, given that we used convenience sampling and the majority were recruited through sickle cell centers and had seen a sickle cell or primary care provider in the previous year. Due to the shortage of adult sickle cell specialists in the U.S., most adults with SCD do not have access to needed preventive care. The impact of disparities in access to care on HRQoL can only be determined when more patients who are “unaffiliated” with SCD care are recruited into research. Barriers to health care access were reported in less than 20% of our study population and this may be an underestimation due to selection bias of patients who are already established in specialized SCD centers.

The cross-sectional nature of the study precludes any conclusions about causal relations between study variables and the PROs. To reduce participant burden, we did not include all items for every PRO measure, thereby limiting full comparison with studies using the complete PRO measures. However, these measures have been constructed to maintain precision even when single, or a few items are used. Our registry data collection included both self-report and information extracted from medical records, however for completeness of data, we only used self-reports of SCD complications experienced, and these reports may suffer from recall bias or may not correspond with actual complications. Further, our reliance on self-report data poses the potential risk of subjectivity and interindividual variation.

We did not have data on several potential contributing factors to the PRO measures, such as other mental health symptoms, e.g., anxiety; actual experience of stigma and discrimination; chronic pain; coping and self-efficacy. We acknowledge that our measure of depression is a self-report of “ever received treatment for depression” so the prevalence of “depression” that we found on the order of 26% may be an underestimate. We did not use data on healthcare utilization from the medical record in these analyses given excess missing data. Study limitations notwithstanding, our research contributes to the literature in its examination of inter-relations between modern PRO measures and SCD-related and other variables within a conceptual model and utilizing a large, geographically diverse sample.

## Conclusions

Reliable and valid PRO measurement is essential to the design of clinical trials and other research [58, 59]. Authoritative bodies including the Centers for Medicaid and Medicare Services and the Food and Drug Administration have prioritized the use of PRO measures for clinical and research applications [60, 61]. Results from this study can provide a baseline for longitudinal investigations that can establish sensitivity to change of the PRO measures and advance our understanding of how SCD and its treatments impact outcomes. We highlighted how critical it is to view lives, care and treatments for individuals with SCD within a biopsychosocial model given our sample’s high prevalence of history of depression, impact of pain experiences in every PRO domain, yet positive associations with fewer barriers to care and disease complications and inter-relations between these variables and socio-demographics such as income and employment status. This research supports that PRO measures can provide meaningful information for providers and patients to improve HRQoL, as well as inform multi-dimensional approaches for providing more effective interventions to improve outcomes.

## Supplementary Information

Below is the link to the electronic supplementary material.

Supplementary file1 (DOCX 50 kb)

## Data Availability

All data and materials support our published claims and comply with field standards.
