# Supplementary material for: An evaluation of patient-reported outcomes in sickle cell disease within a conceptual model
Source: Qual Life Res. 2022 Apr 21;31(9):2681–94. doi: 10.1007/s11136-022-03132-z (PMC9356962; doi:10.1007/s11136-022-03132-z)
Supplement: Supplementary file 1 — Supplementary file1 (DOCX 50 kb) [file 11136_2022_3132_MOESM1_ESM.docx]

| **Supplemental Table 1. ASCQ-Me Measures- Demographics and Clinical Characteristics** | | | | | |  |  |  |  |  |  |  |
| --- | --- | --- | --- | --- | --- | --- | --- | --- | --- | --- | --- | --- |
|  |  |  |  |  |  |  |  |  |  |  |  |  |
|  | **Emotional Impact** | | | **Social Functioning Impact** | | | **Pain Impact** | | | **Sleep Impact** | | |
|  | **(n = 1530; EI<40 = 200)** | | | **(n=1924, SF<40 n = 281)** | | | **(n=1885, PI<40 n = 410)** | | | **(n=1598, SI<40 n = 270)** | | |
| **Variables** | **<40** | **>40** | **p-value** | **<40** | **>40** | **p-value** | **<40** | **>40** | **p-value** | **<40** | **>40** | **p-value** |
| **Age, n (%)** |  |  |  |  |  |  |  |  |  |  |  |  |
| 18-24 | 58 (22.7 | 574 (32.7 | 0.0006 | 77 (25.7) | 559 (32.3) | 0.06 | 102 (23.3) | 535 (33.6) | <0.0001 | 102 (30) | 532 (31.8) | 0.2 |
| 25-34 | 127 (49.8%) | 748 (42.6%) |  | 146 (48.7) | 740 (42.7) |  | 201 (46.0%) | 681 (42.8%) |  | 162 (47.6) | 713 (42.6) |  |
| 35+ | 70 (27.5 | 434 (24.7 |  | 77 (25.7) | 432 (25) |  | 134 (30.7) | 376 (23.6) |  | 76 (22.4) | 428 (25.6) |  |
| **Gender Identity Male, n (%)** | 78 (30.6 | 788 (44.9) | <0.0001 | 100 (33.3) | 777 (44.9) | **0.0002** | 167 (38.2) | 709 (44.5) | 0.02 | 126 (37.1) | 742 (44.4) | **0.01** |
| **Education, n (%)** |  |  |  |  |  |  |  |  |  |  |  |  |
| <High School | 28 (11.1) | 176 (10.1) | 0.6 | 34 (11.5) | 173 (10.1) | 0.04 | 57 (13.4) | 150 (9.5) | 0.02 | 44 (13.1) | 161 (9.7) | 0.08 |
| High school | 81 (32) | 526 (30.3) |  | 106 (35.8) | 504 (29.5) |  | 135 (31.8) | 469 (29.9) |  | 100 (29.8) | 506 (30.6) |  |
| Some college | 92 (36.4) | 606 (34.9) |  | 101 (34.1) | 604 (35.3) |  | 151 (35.5) | 552 (35.1) |  | 125 (37.2) | 570 (34.5) |  |
| Ref: College/Advanced | 52 (20.6) | 426 (24.6) |  | 55 (18.6) | 429 (25.1) |  | 82 (19.3) | 400 (25.5) |  | 67 (19.9) | 415 (25.1) |  |
| **Income, n (%)** |  |  |  |  |  |  |  |  |  |  |  |  |
| $25,000 and under | 160 (68.1) | 820 (52.4) | <0.0001 | 185 (68.3) | 807 (52.2) | <.0001 | 257 (66.2) | 725 (51.2) | <0.0001 | 202 (65.4) | 777 (52.1) | <0.0001 |
| $25,001 - $50,000 | 40 (17) | 358 (22.9) |  | 43 (15.9) | 358 (23.2) |  | 70 (18) | 329 (23.2) |  | 65 (21) | 332 (22.3) |  |
| Ref: $50,001+ | 35 (14.9) | 386 (24.7) |  | 43 (15.9) | 381 (24.6) |  | 61 (15.7) | 363 (25.6) |  | 42 (13.6) | 381 (25.6) |  |
| **Employment, n (%)** |  |  |  |  |  |  |  |  |  |  |  |  |
| Ref: Working | 60 (23.7) | 680 (39.4) | <0.0001 | 51 (17.2) | 694 (40.8) | <0.0001 | 91 (21.4) | 652 (41.7) | <0.0001 | 92 (27.5) | 647 (39.3) | <0.0001 |
| Disabled | 99 (39.1) | 396 (22.9) |  | 132 (44.4) | 370 (21.7) |  | 171 (40.1) | 325 (20.8) |  | 105 (31.3) | 392 (23.8) |  |
| Student | 28 (11.1) | 243 (14.1) |  | 30 (10.1) | 241 (14.2) |  | 36 (8.5) | 235 (15) |  | 36 (10.7) | 243 (14.2) |  |
| Other | 66 (26.1) | 409 (23.7) |  | 84 (28.3) | 397 (23.3) |  | 128 (30) | 351 (22.5) |  | 102 (30.4) | 373 (22.7) |  |
| **Marital status , n (%)** |  |  |  |  |  |  |  |  |  |  |  |  |
| Married/Living as married | 42(16.8) | 268(16.2) | 0.046 | 43(14.8) | 265(16.3) | 0.3 | 73(17.5) | 239(16.0) | 0.07 | 44(13.5) | 267(16.9) | 0.3 |
| Divorced/Separated/Widow | 24(9.6) | 93(5.6) |  | 24(8.2) | 96(5.9) |  | 35(8.4) | 83(5.6) |  | 23(7.1) | 94(6.0) |  |
| Ref: Never married | 184(73.6) | 1290(78.1) |  | 224(77.0) | 1268(77.8) |  | 310(74.2) | 1171(78.4) |  | 258(79.4) | 1217(77.1) |  |
| **Diabetes "No", n (%)** | 237 (95.6) | 1,678 (97.6) | 0.06 | 281 (95.6) | 1,652 (97.6) | 0.05 | 412 (96.0) | 1518 (78.4) | 0.053 | 327 (97.6) | 1,589 (97.3) | 0.8 |
| **Ever treated for depression "No", n (%)** | 124 (51.2) | 1,308 (77.2) | <0.0001 | 168 (59.4) | 1,278 (76.4) | <0.0001 | 273 (66.6) | 1163 (75.9) | 0.0001 | 184 (57.3) | 1,246 (77.1) | <0.0001 |
| **SCD dx, n (%)** |  |  |  |  |  |  |  |  |  |  |  |  |
| Ref: SS/Sβ^0^ Thalassemia | 183 (72) | 1,277 (72.7) | 0.7 | 220 (73.3) | 1,253 (72.4) | 0.7 | 318 (72.8) | 1,152 (72.4) | 0.8 | 244 (72) | 1,216 (72.7) | 0.9 |
| SC | 52 (20.5) | 371 (21.1) |  | 59 (19.7) | 369 (21.3) |  | 89 (20.4) | 340 (21.4) |  | 72 (21.2) | 354 (21.2) |  |
| **Disease Modifying Therapies "yes", n (%)** | 176 (70.1) | 1103 (63.8%) | 0.05 | 188 (64.2) | 1,102 (64.7) | 0.9 | 290 (68.6) | 999 (63.5) | 0.05 | 225 (67.6) | 1,053 (64) | 0.2 |
| **# of Access/ Accommodations/Insurance barriers to care, n (%)** |  |  |  |  |  |  |  |  |  |  |  |  |
|  | 77 (30.2) | 284 (16.2) | <0.0001 |  |  | <.0001 |  |  | 0.0002 |  |  | < .0001 |
| 1 or more (vs 0) | 42 (16.5 | 126 (7.2 |  | 83 (27.7) | 285 (16.5) |  | 106 (24.3) | 262 /16.5) |  | 93 (27.4) | 269(16.1) |  |
| **# of individual barriers to care** |  |  |  |  |  |  |  |  |  |  |  |  |
|  |  |  | <0.0001 |  |  | <0.0001 |  |  | 0.04 | 88 (25.9) | 277 (16.6) | 0.003 |
| 1 or more (vs 0) | 86 (33.7) | 277 (15.8) |  | 87 (29) | 285 (16.5) |  | 94 (21.5) | 275 (17.3) |  | 31 (9.1) | 84 (5) |  |
| **ASCQ-Me Pain episodes frequency score** |  |  |  |  |  |  |  |  |  |  |  |  |
| Mean(SD) | 54.6 (8.2) | 48.4 (11.2) | <0.0001 | 54.3 (8.3) | 48.4 (11.2) | <0.0001 | 55.7 (7.8) | 47.4 (11.1) | <0.0001 | 52.5 (9.3) | 48.4 (11.2) | <0.0001 |
|  |  |  |  |  |  |  |  |  |  |  |  |  |
| **ASCQ-Me Pain episodes severity score** |  |  |  |  |  |  |  |  |  |  |  |  |
| Mean (SD) | 55.1 (7.3) | 50.2 (9.8) | <0.0001 | 55.9 (7.3) | 50 (9.7) | <0.0001 | 55.8 (6.5) | 49.5 (9.9) | <0.0001 | 53.2 (8.8) | 50.3 (9.8) | <0.0001 |
|  |  |  |  |  |  |  |  |  |  |  |  |  |
| **Expanded ASCQ-Me SCD-MHC score - 13 items*** |  |  |  |  |  |  |  |  |  |  |  |  |
| Low (0-1) | 29 (12.6) | 392 (24.1) | <0.0001 | 27 (9.7) | 397 (24.9) | <0.0001 | 51 (13) | 371 (25.2) | <0.0001 | 40 (12.8) | 382 (24.7) | <0.0001 |
| Medium (2-3) | 59 (25.5) | 660 (40.6) |  | 107 (38.4) | 617 (38.8) |  | 126 (32.1) | 597 (40.5) |  | 107 (34.2) | 613 (29.7) |  |
| High (>3) | 143 (61.9) | 573 (35.3) |  | 145 (52) | 578 (36.3) |  | 215 (54.8) | 507 (34.4) |  | 166 (53) | 549 (35.6) |  |

| **Supplemental Table 2. Neuro-QOL and PROMIS Measures- Demographics and Clinical Characteristics** | | | | | |  |  |  |  |
| --- | --- | --- | --- | --- | --- | --- | --- | --- | --- |
|  |  |  |  |  |  |  |  |  |  |
|  | **Neuro-QOL Cognitive Functioning** | | | **PROMIS Emotional Distress** | | | **PROMIS Fatigue** | | |
|  | **(n=2040, CF<40 = 264)** | | | **(n = 2014; ED>60 = 405)** | | | **(n=2004, FI > 60 = 440)** | | |
| **Variables** | <40 (worse) | >=40 (better) | p-value | >60 (worse) | <= 60 (better) | p-value | >60 (worse) | <= 60 (better) | p-value |
| **Age** |  |  |  |  |  |  |  |  |  |
| 18-24 | 86 (32.6) | 551 (31) | 0.4 | 107 (26.4) | 526 (32.7) | 0.04 | 117 (26.6) | 513 (32.8) | 0.04 |
| 25-34 | 106 (40.2) | 789 (44.4) |  | 183 (45.2) | 693 (43.1) |  | 202 (45.9) | 670 (42.8) |  |
| 35+ | 72 (27.3) | 436 (24.5) |  | 115 (28.4) | 390 (24.2) |  | 121 (27.5) | 381 (24.4) |  |
| **Gender Identity Male** | 91 (34.5) | 794 (44.7) | 0.002 | 152 (37.5) | 716 (44.5) | 0.01 | 107 (24.3) | 756 (48.3) | <0.0001 |
| **Education** |  |  |  |  |  |  |  |  |  |
| <High School | 32 (12.3) | 176 (10) | 0.09 | 49 (12.3) | 155 (9.7) | 0.03 | 39 (9) | 165 (10.7) | 0.003 |
| High school | 86 (33.1) | 526 (30) |  | 113 (28.3) | 494 (31.1) |  | 107 (24.6) | 498 (32.2) |  |
| Some college | 95 (36.5) | 615 (35) |  | 157 (39.3) | 542 (34.1) |  | 163 (37.5) | 530 (34.3) |  |
| Ref: College/Advanced | 47 (18.1) | 438 (25) |  | 80 (20.1) | 399 (25.1) |  | 126 (29) | 352 (22.8) |  |
| **Income** |  |  |  |  |  |  |  |  |  |
| $25,000 and under | 158 (65.8) | 838 (52.9) | 0.0002 | 251 (67.5) | 730 (51) | <0.0001 | 222 (56.3) | 752 (53.8) | 0.4 |
| $25,001 - $50,000 | 48 (20) | 355 (22.4) |  | 67 (18) | 331 (23.1) |  | 77 (19.5) | 320 (22.9) |  |
| Ref: $50,001+ | 34 (14.2) | 392 (24.7) |  | 54 (14.5) | 369 (25.8) |  | 95 (24.1) | 326 (23.3) |  |
| **Employment** |  |  |  |  |  |  |  |  |  |
| Ref: Working | 69 (26.4) | 678 (38.8) | 0.0003 | 110 (27.6) | 631 (39.8) | <0.0001 | 150 (34.6) | 588 (38.2) | 0.1 |
| Disabled | 88 (33.7) | 418 (23.9) |  | 143 (35.8) | 353 (22.3) |  | 118 (27.2) | 376 (24.4) |  |
| Student | 39 (14.9) | 233 (13.3) |  | 44 (11) | 227 (14.3) |  | 51 (11.8) | 220 (14.3) |  |
| Other | 65 (24.9) | 417 (23.9) |  | 102 (25.6) | 373 (23.5) |  | 115 (26.5) | 356 (23.1) |  |
| **Marital status** |  |  |  |  |  |  |  |  |  |
| Married/Living as married | 32(13.0) | 281(16.7) | 0.2 | 60(15.6) | 251(16.5) | 0.1 | 83(19.4) | 223(15.2) | 0.06 |
| Divorced/Separated/Widow | 12(4.9) | 108(6.4) |  | 33(8.6) | 85(5.6) |  | 30(7.0) | 86(5.9) |  |
| Ref: Never married | 203(82.2) | 1292(76.9) |  | 292(75.8) | 1182(77.9) |  | 314(73.5) | 1158(78.9) |  |
| **Diabetes "No"** | 247 (96.5) | 1,695 (97.5) | 0.4 | 379 (95.9) | 1,538 (97.7) | 0.06 | 416 (96.3) | 1,493 (97.6) | 0.12 |
| **Ever treated for depression "No"** | 148 (59) | 1,305 (76.2) | <.0001 | 193 (50) | 1,241(79.9) | <0.0001 | 256 (61.1) | 1,171 (77.4) | <.0001 |
| **SCD dx** |  |  |  |  |  |  |  |  |  |
| Ref: SS/Sβ^0^ Thalassemia | 194 (73.5) | 1,288 (72.6) | 0.6 | 287 (70.9) | 1,173 (72.9) | 0.1 | 297 (67.5) | 1,155 (73.9) | 0.02 |
| SC | 57 (21.6) | 372 (21) |  | 83 (20.5) | 342 (21.3) |  | 113 (25.7) | 310 (19.8) |  |
| **Disease Modifying Therapies "yes"** | 175 (68.4) | 1,124 (64.2) | 0.2 | 260 (65.3) | 1,020 (64.4) | 0.7 | 258 (59.9) | 1,015 (65.8) | 0.02 |
| **# of Access/Accommodations/Insurance barriers to care** |  |  |  |  |  |  |  |  |  |
|  |  |  | <0.0001 |  |  | <0.0001 |  |  | <0.0001 |
| 1 or more (vs 0) | 78 (29.5) | 293 (16.5) |  | 118 (29.1) | 244 (15.2) |  | 125 (28.4) | 237 (15.2) |  |
| **# of individual barriers to care** |  |  |  |  |  |  |  |  |  |
| Ref: 0 |  |  | < .0001 |  |  | <0.0001 |  |  | <.0001 |
| 1 or more | 77 (29.2) | 293 (16.5) |  | 123 (30.4) | 241 (15.0) |  | 51 (11.6) | 63 (4) |  |
| **ASCQ-Me Pain episodes frequency score** |  |  |  |  |  |  |  |  |  |
| Mean(SD) | 51.7 (9.8) | 48.9 (11.2) | <0.0001 | 52.8 (9.8) | 48.3 (11.2) | <.0001 | 51.7 (10.2) | 48.5 (11.2) | <0.0001 |
|  |  |  |  |  |  |  |  |  |  |
| **ASCQ-Me Pain episodes severity score** |  |  |  |  |  |  |  |  |  |
| Mean (SD) | 51.6 (8.9) | 50.7 (9.7) | 0.2 | 52.5 (8.4) | 50.4 (9.9) | <0.0001 | 52.7 (8.5) | 50.2 (9.9) | <.0001 |
|  |  |  |  |  |  |  |  |  |  |
| **Expanded ASCQ-Me SCD-MHC score - 13 items*** |  |  |  |  |  |  |  |  |  |
| Low (0-1) | 33 (13.8) | 392 (23.9) | <0.0001 | 53 (14.4.) | 369 (24.8) | <0.0001 | 82 (20.1) | 337 (23.4) | 0.008 |
| Medium 2-(3) | 86 (35.8) | 642 (39.1) |  | 114 (30.9) | 606 (40.7) |  | 142 (34.8) | 577 (40) |  |
| High (>3) | 121 (50.4) | 606 (37) |  | 202 (54.7) | 514 (34.5) |  | 184 (45.1) | 527 (36.6) |  |
